# Lessons From Deep Neural Networks for Studying the Coding Principles of Biological Neural Networks

**DOI:** 10.3389/fnsys.2020.615129

**Published:** 2021-01-15

**Authors:** Hyojin Bae, Sang Jeong Kim, Chang-Eop Kim

**Affiliations:** ^1^Department of Physiology, Gachon University College of Korean Medicine, Seongnam, South Korea; ^2^Laboratory of Neurophysiology, Department of Physiology, Seoul National University College of Medicine, Seoul, South Korea

**Keywords:** deep neural networks, biological neural networks, systems neuroscience, shortcut learning, neural coding, neural feature

## Abstract

One of the central goals in systems neuroscience is to understand how information is encoded in the brain, and the standard approach is to identify the relation between a stimulus and a neural response. However, the feature of a stimulus is typically defined by the researcher's hypothesis, which may cause biases in the research conclusion. To demonstrate potential biases, we simulate four likely scenarios using deep neural networks trained on the image classification dataset CIFAR-10 and demonstrate the possibility of selecting suboptimal/irrelevant features or overestimating the network feature representation/noise correlation. Additionally, we present studies investigating neural coding principles in biological neural networks to which our points can be applied. This study aims to not only highlight the importance of careful assumptions and interpretations regarding the neural response to stimulus features but also suggest that the comparative study between deep and biological neural networks from the perspective of machine learning can be an effective strategy for understanding the coding principles of the brain.

## Introduction

A standard approach to study the neural coding principle in biological neural networks (BNNs) is to characterize the statistical properties of neural responses and elucidate their association with sensory or other information (Dayan and Abbott, 2001; Panzeri et al., 2015). For example, one can use statistical tests to compare neural responses for the feature set or decoding models that predict the feature labels from neural activity, revealing the information content present in the brain region.

The use of machine learning (ML) in neuroscience has grown rapidly during the last decade (Glaser et al., 2019). The role of ML in neuroscience ranges from a tool for neural data analysis (Carlson et al., 2013; Lebedev et al., 2014; Mathis et al., 2018; Pandarinath et al., 2018) to a model for the brain (Cadieu et al., 2014; Yamins et al., 2014; Kell et al., 2018; Keshishian et al., 2020; Yang and Wang, 2020). In particular, it has become a popular idea that deep neural networks (DNNs) can serve as a good model of biological networks considering their near human-level performance across challenging domains (Marblestone et al., 2016; Cichy and Kaiser, 2019). Although it has been pointed out that DNNs lack biological plausibility and are not transparent, remarkable developments have been made enabling one to analyze and understand their representation (Samek et al., 2016; Fong and Vedaldi, 2018; Zhou et al., 2018; Cohen et al., 2019; Zhang et al., 2019), and recent studies propose that DNN models can provide insights for the brain's computing mechanism based on their similar response properties (Kriegeskorte, 2015; Güçlü and van Gerven, 2017; Kell and McDermott, 2019). As they share the question of understanding the representation of the neural networks, there are opportunities for synergy between the DNN enabling controllable and tractable simulation and the BNN with significantly greater experience in the matter (Barrett et al., 2019; Richards et al., 2019)

In this study, we demonstrate the dangers latent in the widely used research framework for identifying informational content from a neural representation. It is noteworthy that these dangers are related to the problems that have been raised in DNN research, which has rich experience in dealing with the so-called black box. In particular, we focus on revealing misleading points that may arise from the researcher-defined feature space. By employing the DNN as an *in silico* model of the BNN, we simulate four likely scenarios and present BNN studies to which our points can be applied as follows. (A) Owing to the inaccessibility of the full feature space, a researcher can misjudge the neural feature selectivity. (B) The researcher-defined feature might be a confounding variable coupled with the ground truth feature and neural response. (C) Overlooking the inherent assumption for the feature space of the decoding model may result in an overestimation of the network feature representation. (D) Misassumptions regarding the feature complexity or disregarding the internal state coding may result in an overestimation of the noise correlation. Finally, we discuss the root cause of constraints in identifying the association between the predefined feature and the neural response and suggest the feasibility of a comparative approach between DNNs and BNNs.

## Results

### The Deep Neural Network Trained on CIFAR-10 as a Model of BNN

**Figure 1:**
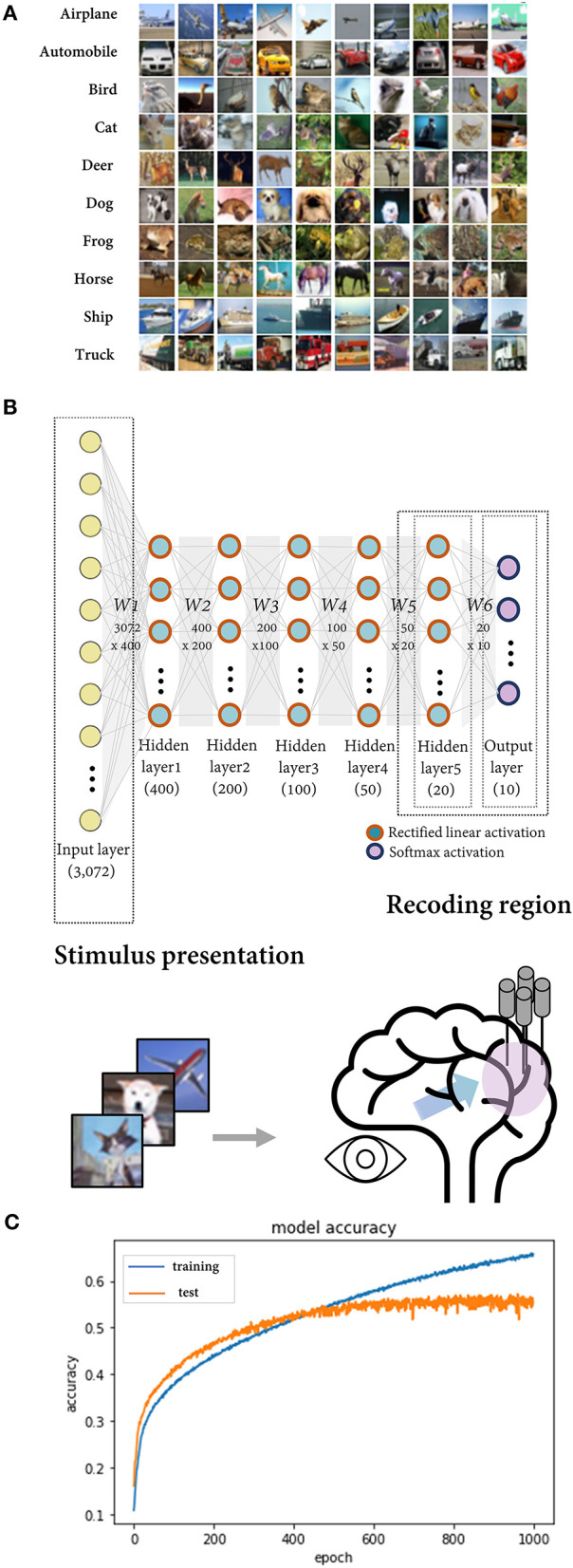
The deep neural network trained on CIFAR-10 as a model of the biological neural network. **(A)** The CIFAR-10 dataset consisting of 32 × 32 × 3 images in 10 classes. **(B)** Deep neural network composed of six layers with dense connections. The rectified linear activation function was implemented for the hidden layers and the softmax function for the output layer. The output layer (in scenarios 1, 2, and 4) or the last hidden layer (in scenario 3) was regarded as the region of interest in the simulation, and their activations for the input image vector correspond to recorded neural activity upon sensory stimuli presentation. **(C)** After ~500 epochs of training with a batch size of 512, the model showed a saturated accuracy on test set data at ~53% (baseline performance = 10%). The blue and orange lines represent the model performance for the training set and the test set, respectively.

To simulate potential errors in a neural coding study, we mimic a BNN by using a DNN model trained on an image dataset. A six-layer feedforward fully connected neural network was constructed and trained using the CIFAR-10 dataset, which comprises 60,000 images in 10 classes (airplane, automobile, bird, cat, deer, dog, frog, horse, ship, and truck) (Figures 1A,B). After 500 epochs of training using stochastic gradient descent with a batch size of 512, the model demonstrated a saturated test set accuracy of ~53% (chance-level performance = 10%) (Figure 1C). It is known that convolutional neural networks (CNNs) perform better for image data. Nonetheless, since we intended to make DNN serve as a model of BNN at the general level rather than to confine it as a model for the visual processing of the brain, we employed the fully connected network, which is the most fundamental architecture of artificial neural networks.

The input image, image class, and model output correspond to the stimulus presented in the experiment, the feature defined by the researcher, and the recorded neural activity, respectively. Ten units (nodes) in the output layer calculate the probability of each class for the input image. They are regarded as neural units tuned to each of the 10 classes like neurons in the inferior temporal cortex that selectively respond to complex visual stimuli such as faces (Bruce et al., 1981). For convenience, we refer to them as their preferred class (e.g., the airplane unit). In the case of the DNN model, the genuine feature space is predefined as labels of the training data. Using this model, we demonstrate the possible errors that may occur when a researcher investigates neural coding principles while presenting a prepared feature set. The output layer, or the last hidden layer of the model, corresponds to the recorded brain region in the simulation for scenarios 1, 2, and 4 and scenario 3 (Figure 1B).

### Scenario 1. Suboptimal Feature Selectivity

**Figure 2:**
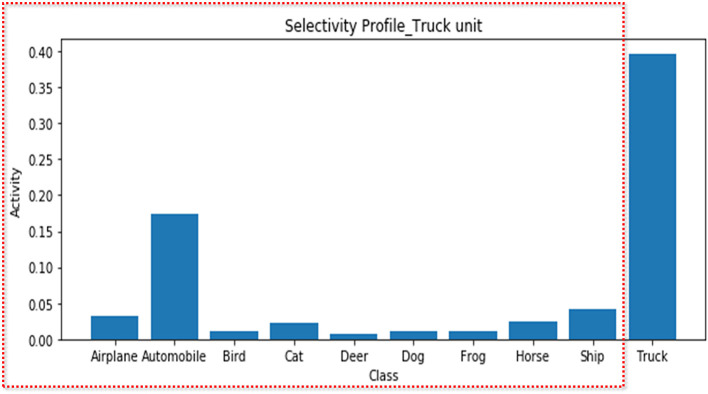
Selectivity profile of the truck unit. The average activities of the truck unit for each stimulus feature are presented. Although the truck unit is a population of neurons tuned to the truck feature, if only the features in the red dotted box are presented, the suboptimal feature (automobile) may appear to be the variable that best accounts for the unit's activity.

In the first scenario, we demonstrate a possible error when determining neural feature selectivity by the differences in the responses of the neural unit to the presented feature set. To simulate a situation in which a researcher records a neuron that is highly tuned on the truck feature and explores the feature selectivity, we averaged the response of the truck unit to each class while showing the test set images of all classes. As expected, this unit exhibited selectivity for the truck feature [the regression coefficient and explained variance were 0.55 (*p* < 0.001) and 0.61, respectively] (Figure 2). However, if the researcher had not included the truck images in the stimulus set, only the results for classes excluding trucks (red box in Figure 2) would have been obtained, and from this, the researcher would have been apt to conclude that the truck unit responds selectively to the automobile feature [the regression coefficient and explained variance were 0.21 (*p* < 0.001) and 0.30, respectively]. In fact, experimental settings wherein a researcher determines the feature selectivity based on the neural response to the features presented in the experiment are common in practice (Miyashita and Chang, 1988; Liu and Richmond, 2000; Tanaka et al., 2001; Stalnaker et al., 2010).

Indeed, recognition of the limitations inherent in the experimental settings was also found in previous studies. Sauerbrei et al. (2015) measured motor-related variables (speed, acceleration, roll, pitch, and electromyogram) in freely moving mice to reveal the information represented in the variability of Purkinje cell activity during locomotion. It was discussed that the measurable variables in freely moving mice that were not anesthetized without fixing the head were limited, and hence, the possibility of primary variables other than the measured features could not be eliminated (Sauerbrei et al., 2015). Another investigation demonstrated that the internal state (satiety) was encoded in global brain regions, and the propagation of the sensory information was gated according to the animal's satiety state. However, the authors recognized the possibility that variables such as arousal (which is correlated with satiety) may be more influential than satiety (Allen et al., 2019).

There are studies in which the raised concerns have been demonstrated more directly. While primary visual cortex neurons have traditionally been assumed to primarily encode the local orientation components of high-order patterns, large-scale two-photon imaging of the primary visual cortex neurons combined with an extensive set of stimuli in awake macaques demonstrated that a large portion of neurons in the superficial layer of the primary visual cortex exhibited high selectivity to various complex patterns, such as curvatures, corners, junctions, and other higher-order patterns (Tang et al., 2018). Furthermore, even for neurons selective to a high-level complex pattern, most of them showed significant tuning for orientation. Hence, they reported that our understanding of neural selectivity may be biased and restricted depending on the neurons that can be sampled and the stimuli that can be evaluated.

Obviously, it is impossible to predefine the entire feature space of the recording region. Moreover, physically measurable variables are limited depending on the experimental settings. Nevertheless, it is necessary to recognize these inevitable constraints, and both the researcher's description and the reader's interpretation must be carefully addressed. In other words, the features that are coupled with the neural response cannot be guaranteed to be the optimal feature, and the definitions of feature selectivity are premised on the specific experimental setting. Therefore, careful interpretations of readers considering the context (i.e., the defined feature space in the experiment) and a rigorous description by the researchers are required.

### Scenario 2. Irrelevant Feature Selectivity

**Figure 3:**
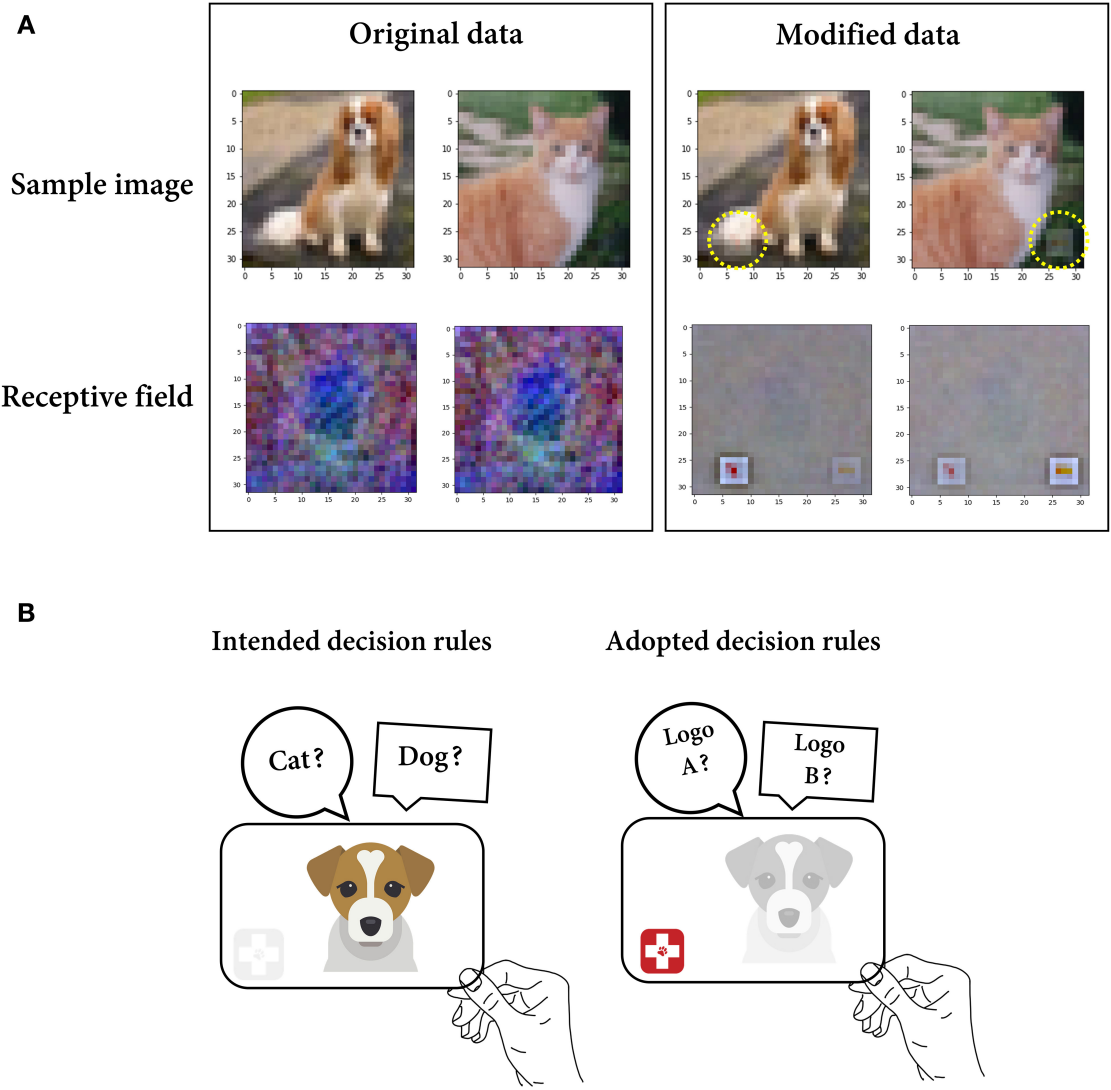
Irrelevant feature selectivity. **(A)** Instance images (top) of a dog and cat in the original/modified (logo-synthesized) dataset and reconstructed receptive field (bottom) for the dog and cat classes from the model trained on either the original or modified dataset. **(B)** A schematic figure illustrating the situation where the solution learned by the model (right) is different from the researcher's intended solution (left).

In the second scenario, we simulate a case where the feature defined by the researcher was a confounding variable coupled with both the ground truth feature and neuronal responses. To simulate the case where the stimuli presented by the researcher contain features that are difficult to recognize, we modified the data by synthesizing two different veterinary hospital logos in the images of cats and dogs (Figure 3A). Compared with the accuracy for the original data in distinguishing between cats and dogs (38%), the model trained on the dataset containing cat and dog images with a logo demonstrated a much higher classification accuracy of 78%. If the researcher judges only the latter result without recognizing the existence of logos, it may be easily concluded that the model encodes a discriminative feature for each class. However, the features that the model encodes are presumably the logo pattern in this case (Figure 3B). To confirm this, we reconstructed the receptive field of each class unit through the weighted summation of the activation values in every route linking a single input pixel and a class unit (see the Materials and Methods section). Consequently, it was clearly shown that the artificially synthesized logos mainly accounted for the activation of the cat/dog unit (Figure 3A). This indicates that the features defined by the researcher (cat and dog) were merely confounding variables.

Recently, a similar problem has been discussed in the deep learning field under the name of shortcut learning. In shortcut learning, a trained model shows a strong discrepancy between the intended and actual learning strategies, leading to poor generalization to unseen domains. For instance, in a classification task, a DNN model can employ the background context (shortcut feature) as the decision rule to recognize the primary object. It may seem to be accurate for objects in a “common” context (e.g., a cow in a pasture) but unexpectedly fail on objects in an uncommon context (e.g., a cow on the beach) (Supplementary Figure 1) (Szegedy et al., 2013; Beery et al., 2018; Zech et al., 2018). This suggests the problem of understanding the behavior of DNNs based on the researcher's intended solution (Geirhos et al., 2020). It may provide an interesting frame of reference for thinking about shortcut learning for BNN research. That is, even if the recorded neurons respond differently to the presented stimuli, the stimulus feature defined by the researcher is possibly a confounding variable that differs from the actual feature encoded by the BNN.

Indeed, Kim et al. (2019) demonstrated that characterizing the feature selectivity of primary sensory cortex neurons can be misled by describing neurons with a single stimulus feature, disregarding other aspects of sensory stimuli. In many cases, neurons in the primary sensory cortex are classified as non-nociceptive, nociceptive, or convergent neurons, according to their electrophysiological response to innocuous brush stroke and noxious forceps pinch stimuli. Contrary to previously known results, the majority of neurons that appeared to encode noxiousness showed high selectivity for the texture of the stimuli and low selectivity for noxiousness. This implies that the texture is more likely to be the explanatory feature and that noxiousness only resulted in a different response owing to the paired texture. Overall, it should be noted that there may be an alternative cause that can explain the results obtained in the given experimental paradigm.

### Scenario 3. Overestimation of Network Feature Representation

In the third scenario, we demonstrate a potential error when studying the feature representation of the network based on neural decoding. One of the ways to evaluate whether particular information is present in a brain region is to implement decoding models, including ML models, and see if the model can decode features from neural activity with performance above the chance level (Yan et al., 2014; Kriegeskorte and Douglas, 2019). It is important to note that chance-level performance is dependent on the number of labels provided to the decoder. However, it is often overlooked that the entire set of labels represented in the recording region is unavailable, meaning that a genuine baseline performance is beyond our reach. In other words, the decoding performance should be interpreted with the baseline performance, but since a genuine baseline performance cannot be known, the experimental results are prone to being misinterpreted.

**Figure 4:**
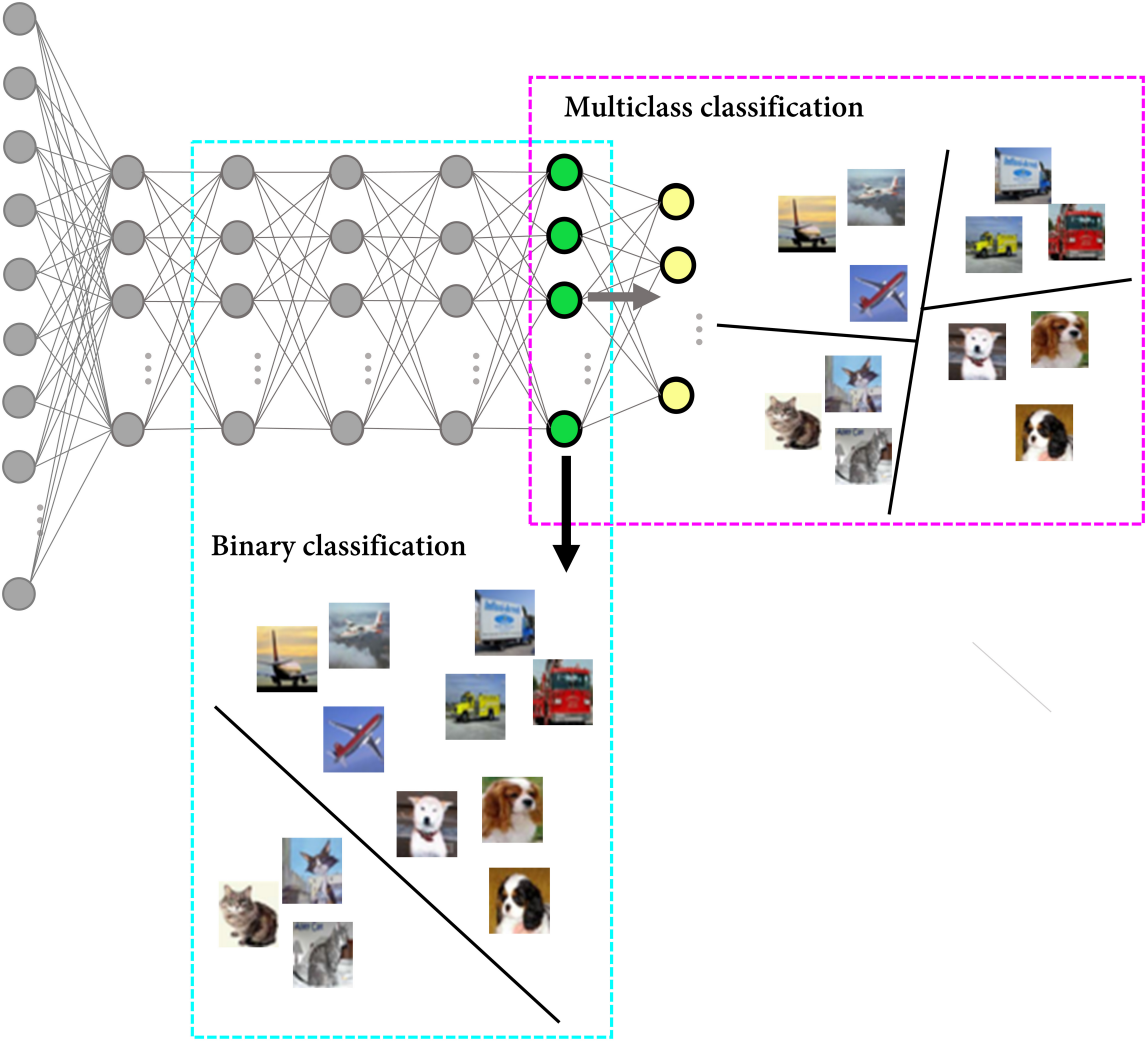
Overestimation of the network feature representation. A schematic figure to explain the difference in the task difficulty depending on the number of exposed labels during model training. While predicting the label of cat images is a multiclass classification problem with 10 class candidates for the DNN model (only 4 classes are presented in the figure for visualization purposes), it is a binary classification task for the linear SVM.

Specifically, we simulate a situation of evaluating whether the cat feature is represented in the recording region using a linear support vector machine (SVM) as the decoder. It is commonly believed that linear decodability is considered evidence for the “explicit” representation in that it can be read by downstream neurons in a single step (Misaki et al., 2010; Kriegeskorte and Kievit, 2013; Ritchie et al., 2019). In this simulation, the last hidden layer of the DNN model was regarded as the region of interest. Since the model was trained on the data with 10 labels (classes), the genuine chance-level performance of the task to classify whether a given stimulus is a cat or not was 10%, and the classification accuracy of the model for the cat class was 28%. Even if this result is declared statistically significant (i.e., the *p-*value falls below the threshold), its effect size, which provides some indication of practical meaningfulness (Benjamin et al., 2018), may not be enough to conclude the linear separability of the two classes in the neuronal representational space. Now let us assume that the researcher implements a binary classifier to investigate whether the cat feature is represented in the recording region. Since a binary classifier is trained on the data with a binary label, only two options are available for the unseen data (i.e., chance-level performance is 50%). When we trained the linear SVM to classify the cat from other classes, the accuracy for the cat test set images was 82%, and linear decodability might be claimed from this result (Figure 4, Table 1). In brief, when evaluating the feature representation of a neural network based on the decoding performance, it can be inflated due to the high chance-level performance of the task defined by the researcher (e.g., binary classification).

**Table 1:** Comparison of the performance of the deep neural network model and the binary decoder.

**Model**	**Accuracy**	**Baseline accuracy**
Deep neural network model	0.28	0.1
Linear support vector machine	0.82	0.5

*Classification accuracy was used as a rule evaluation measure, and it was evaluated on the same test data (1,000 cat images) for both models. Baseline accuracy indicates the probability that the model matches the class of the input image by chance, which is determined by the number of labels contained in the training data*.

Various decoding methods have been used to decipher neural coding principles from the neural activity patterns that are distributed across neurons or cortical regions (Haxby et al., 2014). In particular, multivoxel pattern analysis, a popular analytical technique for analyzing fMRI data, is widely used in comparing how a distributed pattern of activity over multiple voxels differs between task conditions or stimuli (i.e., multivariate pattern classification) (Popov et al., 2018). It is common to implement a linear classifier to the region of interest to decode binary information and make inferences such as the engagement of certain brain areas in specific tasks or the relationship between brain states and informational content. However, as we pointed out in this scenario, the fact that the genuine dimension of the label space of the BNN is unavailable makes room for misinterpretations of the network feature representation. In other words, even if the decoding performance is statistically significant, it should be noted that it is the result of the test based on the chance-level performance assumed by the decoder model. Both the readers and researchers should be cognizant of the exact characteristics of the decoder model and to what extent the model can account.

### Scenario 4. Overestimation of the Noise Correlation

Finally, we simulate a situation where the noise correlation of the network can be overestimated owing to the misassumption in feature complexity or omission of globally coded features. Noise correlation is the degree to which the trial-by-trial variability in responses for an identical stimulus is shared by a pair of neurons. It is different from the signal correlation measured from the responses of a neuron pair for different stimuli, which indicates a similar tuning property (Supplementary Figure 2) (Cohen and Kohn, 2011). Noise correlation is investigated mainly in the context of its relationship with population coding, network architecture, or behavior (Cohen and Maunsell, 2009; Hofer et al., 2011; Sauerbrei et al., 2015; Ruff and Cohen, 2019). Therefore, a biased estimation of noise correlation can generate consecutive errors in their roles in sensory processing or inferences regarding the network connectivity and the mechanisms that produce them.

Specifically, in this scenario, we want to show that signal correlation can be incorporated into the noise correlation due to the researcher's misassumption of features. The activity of the units in the output layer of the DNN model is regarded as the average activity of the homogenous population, and after training, it is deterministic for the same input. Therefore, to mimic noise correlation estimation in BNN research, we constructed a model capable of stochastically generating individual neural activity (see the Materials and Methods section for details).

#### Feature Complexity

It is known that neurons along the ventral pathway of the human brain are tuned to features of different complexity (Riesenhuber and Poggio, 2002; Güçlü and van Gerven, 2015). Unlike lower cortical areas, the preferred feature of a neuron in the higher visual areas is hard to determine (Riesenhuber and Poggio, 2002). Here, we point out that the noise correlation estimation can be overestimated if the researcher incorrectly assumes the feature complexity. If the features defined by the researcher were subdivided into finer features in the region of interest, a signal correlation may be incorporated into the estimated noise correlation. This is because even if the researcher repeats the stimulus that is identical in terms of the coarse feature, neurons will react differently depending on the tuning property of the fine features.

**Figure 5:**
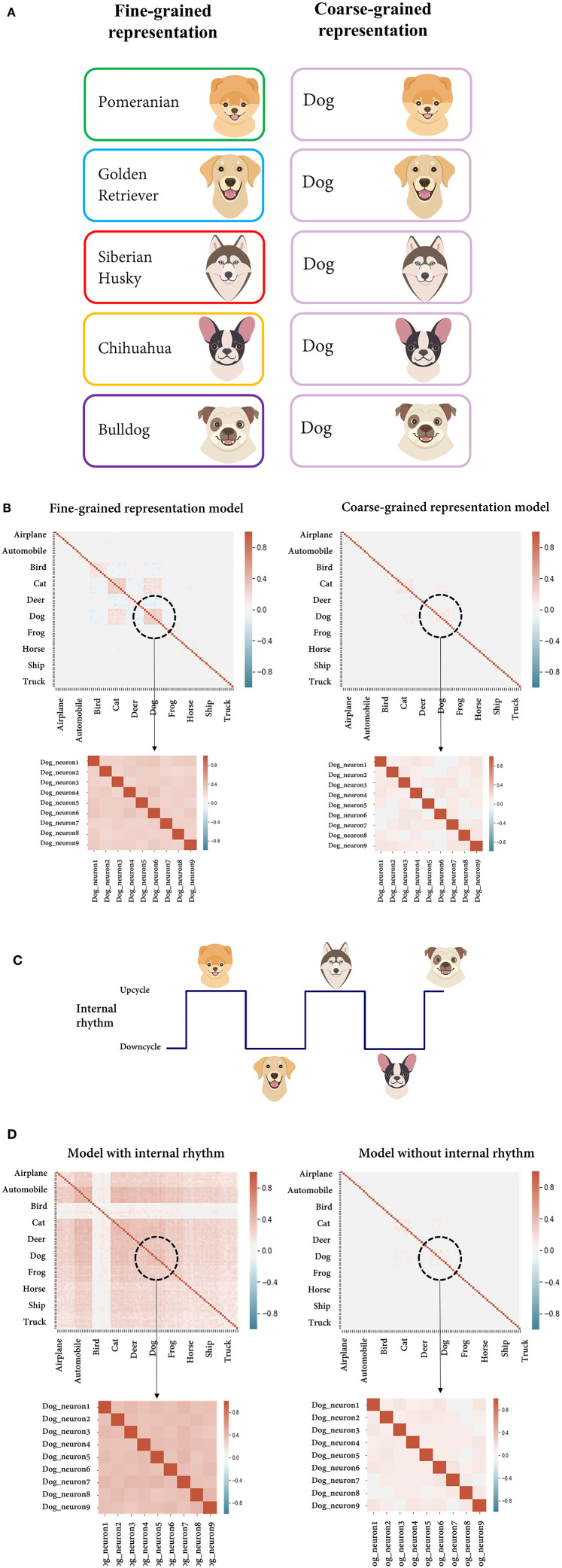
Overestimation of the noise correlation. **(A)** Schematic figure to illustrate the simulated situation in which the feature of a stimulus defined by the researcher is the dog class (right, coarse-grained representation) and the recording region actually encodes the feature at the level of individual dog breeds (left, fine-grained representation). From the perspective of neurons encoding the breeds, dog images of different breeds were recognized as different stimuli, while the researcher regards them as an identical stimulus. **(B)** Correlation matrices of individual neural activities randomly sampled from the model assumed to encode either the dog breed or the dog class (fine-grained/coarse-grained representation model). The enlarged parts are the values from the individual neurons within the dog unit. **(C)** Schematic figure illustrating the simulated situation in which a periodic internal state is globally encoded by the neural population and stimuli are presented alternately in the upcycle and downcycle of the rhythm. **(D)** Correlation matrices of individual neural activities randomly sampled from the model with/without a periodic internal rhythm.

As an example of the scenario, we assumed a situation in which the feature of the stimulus defined by the researcher is the dog class (coarse-grained representation, high-level feature) and the recording region is actually coding the feature at the level of individual dog breeds (fine-grained representation, low-level feature) (Figure 5A). We tried to compare the correlation of the response variability from two models with different feature complexities, each of which accords with the researcher's assumption (coarse-grained representation model) and the actual situation (fine-grained representation model). By differently assuming within-unit variance, the multivariate normal distribution of each model was parameterized, enabling stochastic sampling (see the Materials and Methods section for details). From each model, individual neural activities were randomly sampled by the unit for the images of the dog class consisting of different breeds, and a pairwise correlation was calculated therefrom. From the perspective of the fine-grained representation model, dog images of different breeds (e.g., Pomeranian and golden retrievers) were recognized as different stimuli. Hence, a signal correlation may occur between neurons having a similar preference for the breed. However, from the point of the researcher's view that the region of interest encodes the dog class, dog images are regarded as the same stimulus regardless of the breed. Subsequently, the pairwise correlation calculated from the response variability (which includes signal correlation) is counted solely as noise correlation. Compared with the case where the researcher's hypothesis was correct (coarse-grained representation model), it can be seen that the noise correlation is overestimated (Figure 5B, Table 2).

**Table 2:** Comparison of the estimated noise correlation.

**Model**	**Average correlation**	
	**Total units**	**Dog unit**
Fine-grained model	0.043	0.275
Coarse-grained model	0.029	0.099
Model with internal rhythm	0.215	0.371
Model without internal rhythm	0.028	0.097

*Means of the absolute pairwise correlations calculated from the trial-by-trial variability of neural responses for each conceptual model within scenario 4. The values of the total units are the average of the correlations obtained from the pairs of the 90 total neurons (9 neurons per unit), and the values of the dog unit are the average of the correlations obtained from the pairs between 9 neurons within the dog unit*.

In fact, it is a likely scenario for a researcher to wrongly assume an appropriate level of feature complexity. The feature defined by the researcher could be more coarse-grained or fine-grained than the actual feature, or the tuning property of the feature can change over time. In recent findings, it has been shown that the abstraction level of features varies based on the hierarchy of brain areas, or more abstracted features may emerge after learning within the same population (Connor et al., 2007; Engel et al., 2015; Tang et al., 2018). According to the investigation of Engel et al. (2015) a considerable fraction of lateral intraparietal cortex neurons showed mixed selectivity for both directions and categories, and even feature selectivity was altered after training from pure-directional to pure-category tuning. Therefore, the tuning property defined within the observation point and setting may not be generalized, which can lead to confusion between signal correlation and noise correlation.

#### Internal Dynamics

It is known that local neural activity depends not only on the current sensory input but also on the current brain state (Panzeri et al., 2015), and it has been hypothesized that a significant fraction of trial-to-trial variability of population activity is accounted for by variations in the brain state (Curto et al., 2009). Internal subjective features are difficult to control or directly measure in an experiment, but since they affect the intercorrelation of neurons, they can produce bias in the noise correlation estimation. Here, we simulate the case in which the neglection of the internal state coding leads to the overestimated noise correlation. We mimic the situation in which a periodic internal rhythm is globally represented in the neural network and stimuli are presented alternately in the upcycle and downcycle of the rhythm (Figure 5C). Again, we constructed two different models, either with or without internal rhythm. In the former, after showing 1,000 test set images of dogs to the DNN model, we added artificially generated random values to the outputs of the sampled neurons for half of the instances and subtracted them for the remaining instances. In the latter, individual neural activity for the same input was taken without any manipulation. As expected, compared to the model without internal rhythm, a high correlation in response variability occurred since the model included the signal correlation evoked by jointly coding the internal state (Figure 5D, Table 2). Without considering the internal state feature, it may be regarded as mere noise correlation.

Recent studies have revealed global representation throughout the brain for internal states such as satiety, anxiety, or latent behavioral states. Allen et al. (2019) showed that in thirsty mice, a thirst motivational state (satiety) was globally represented across the brain regions and that the neural responses for the same task-relevant feature were altered as the thirst became gradually satisfied during repeated trials. Stringer et al. (2019) demonstrated representation of behavioral-state information in the primary sensory cortex, suggesting that previously reported trial-by-trial variability during stimulus presentations may depend on it. Another investigation discovered that single prefrontal cortex neurons contribute to complicated cognitive tasks by having mixed selectivity and that they encode internal cognitive processes simultaneously with task-relevant information (Rigotti et al., 2010). Bányai and Orbán (2019) reported that in hierarchical models, inferences for task-related and higher-level perceptual variables were the dual source of noise correlation. Here, we want to emphasize the possibility that signal correlation due to undetected features can be incorporated into the noise correlation.

### Reproducing the Scenarios Using a CNN

**Figure 6:**
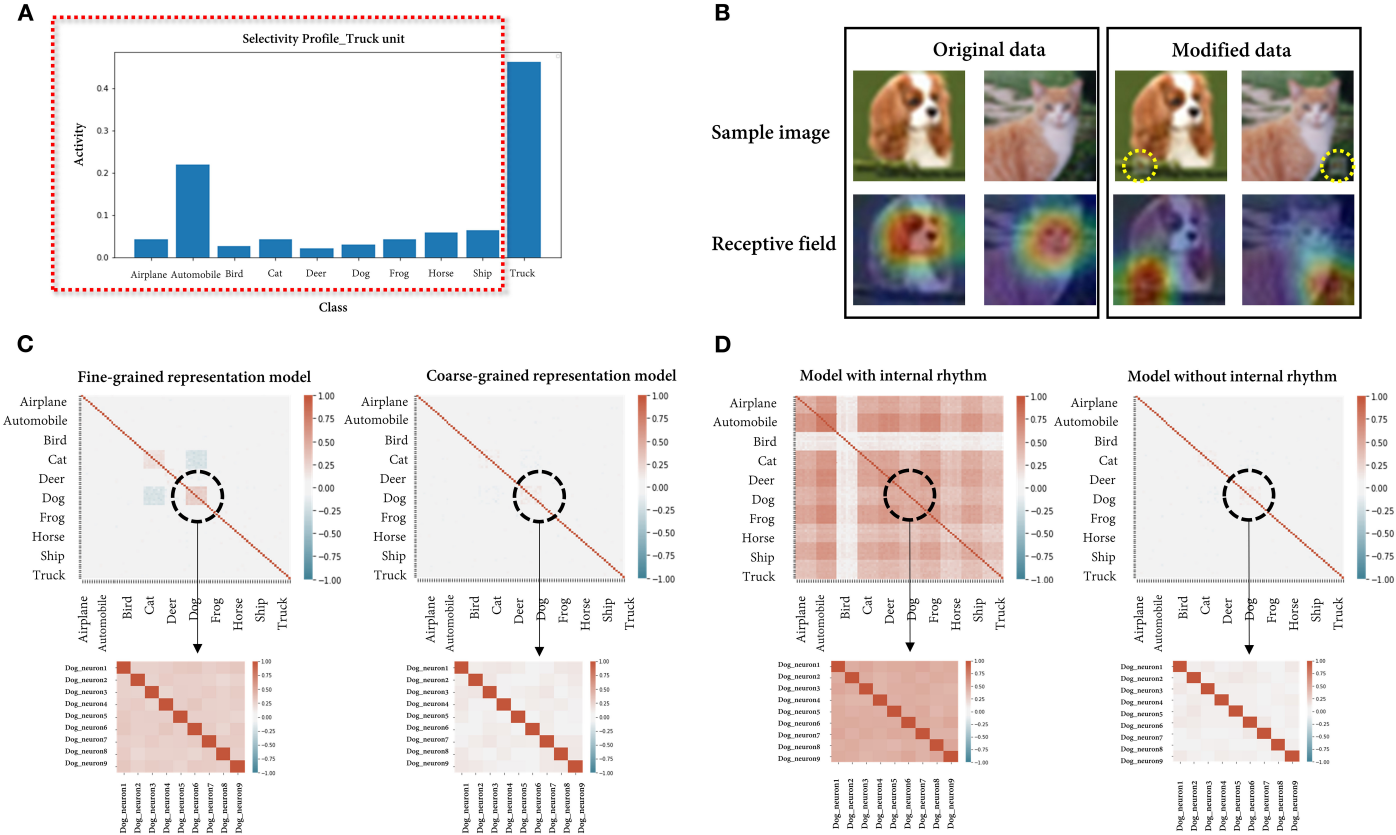
Simulation results using a convolutional neural network. **(A)** The average activities of the truck unit for each stimulus feature are presented. As expected, the unit showed the highest mean activity for the optimal feature (truck), while it also showed considerable activity for a suboptimal feature (automobile). **(B)** Instance images (top) of a dog and cat in the original/modified (logo-synthesized) dataset and the visualized class activation maps (bottom) from the model trained on either the original or modified dataset. The accuracy of the model is 90 and 100% on each dataset, respectively. Note that what the latter model actually sees is the artificially synthesized logo, not the image label (expected feature). **(C)** Correlation matrices of individual neural activities randomly sampled from the model assumed to encode either the dog breed or the dog class (fine-grained/coarse-grained representation model). The enlarged parts are the values from the individual neurons within the dog unit. The averages of the correlations are 0.044 (fine-grained model)/0.038 (coarse-grained model) for the 90 total neurons and 0.360/0.199 for the 9 neurons within the dog unit. **(D)** Correlation matrices of individual neural activities randomly sampled from the model with/without a periodic internal rhythm. The averages of the correlations are 0.407 (model with internal rhythm)/0.038 (model without internal rhythm) for the 90 total neurons and 0.529/0.195 for the 9 neurons within the dog unit.

As mentioned at the beginning, we implemented the fully connected network to avoid limiting the issues raised for the visual system. Nonetheless, given that the examples used are all visual stimuli, we repeated the simulations using a CNN, which is widely used in the field of image processing. After fine-tuning the pretrained ResNet18 (He et al., 2016) model on the same cifar-10 dataset, an accuracy of 94.6% was obtained for the held-out data, and the simulated results were consistent with the previous results in all scenarios (Figure 6). In scenario 3, the classification accuracy of the model for the cat class (85%) and that of linear SVM (91%) did not show much difference since the performance of the model was already saturated.

## Discussion

The primary purpose of this study was to highlight the necessity of the careful inspection of our research framework that identifies the relationship between a stimulus and a neural response based on the predefined feature space. The second aim was to propose the feasibility of bridging the studies of artificial intelligence (AI) and neuroscience in various aspects. In this study, we were able to explicitly demonstrate the misleading points by implementing the DNN model as a BNN model to mimic specific situations in each scenario. This research suggests that the comparative approach between AI and neuroscience can provide new insights into the same problem and can enable alternative interpretations based on accumulated experiences in each field.

This study highlights that the interpretation of empirical results requires care. This is because even if there is an association (dependency) between variables in the observed data, this does not necessarily mean a causal relationship. In other words, associations can arise between variables in the absence of a causal relationship if they have a common cause (Altman and Krzywinski, 2015). As shown in scenarios 1, 2, and 4.2, the oversight of the presence of an unevaluated variable can lead to erroneous conclusions in diverse aspects. Additionally, scenario 3 underlines that the empirical results should be interpreted with the context in which the data were obtained, such as a specific experimental setting and the assumption of the model used. Primarily, the researchers need to describe the experimental conditions in detail and be aware of the extent to which they can infer from the implementation of the techniques (or the models). Even if the statistical significance or the rigor of the experiment is satisfied, deriving the implications from the observation is a different problem. Regarding readers, they tend to focus on the conclusion, thereby missing the detailed contexts. Although it is cognitively demanding to understand the details, a reader's careful attention is needed as much as the researcher's careful reporting.

The 4 scenarios presented in this study basically arise from the fact that the feature of a stimulus is, in fact, an idea defined by the researcher. Selective neurons that encode easily recognizable features, such as orientation, color, and motion, have been found in many brain areas (Kamitani and Tong, 2005). However, unlike the brain regions receiving the primary sensory information, it has been revealed that it is difficult to determine the selective feature in regions performing higher cognitive functions (Poldrack, 2006). Moreover, as bottom-up inputs are integrated, the receptive field may be dynamically modulated by attention in the downstream, making them no longer under the researcher's control (David et al., 2008; Zirnsak and Hamker, 2010; Ibos and Freedman, 2014, 2016; Pattadkal et al., 2018; Wutz et al., 2018). In addition, the vast amount of information that will be addressed at the unconscious level is not even in an interpretable form (György Buzsáki, 2019). A more fundamental concern is that the identification of the informational content of a given representation does not necessarily mean that the representation stands for it (Brook and Akins, 2005; Morgan and Piccinini, 2018; Gomez-Marin and Ghazanfar, 2019). Furthermore, Buzsáki referred to the framework taking invented terms (defined features of the stimulus) as the to-be-explained independent categories and looking for brain mechanisms that can explain those ideas as an “outside-in framework” and claimed that the thing to-be-explained should be the activities of the brain, not the invented terms (“inside-out framework”) (György Buzsáki, 2019).

It is noteworthy to mention that this study is inspired by the failure experiences of DNNs. With many cases where DNNs fail unexpectedly in real-world scenarios, the need to understand the representations they learn has come to the forefront under the name of shortcut learning (Nguyen et al., 2015; Beery et al., 2018; Geirhos et al., 2020). A shortcut strategy refers to the learned decision rule of the model that differs from the researcher's intended solution. While superficially successful, it can no longer be generalized if the model confronts unseen data from different distributions. Shortcut learning in DNNs demonstrates the risk of assigning underlying abilities to the models based on the researcher's assumption (i.e., the expected solution based on how a human would solve the problem). Given that our understanding of the internal mechanism of the brain is still rudimentary, it would be fruitful to learn from mistakes in DNNs.

Our study is based on the idea that DNNs can serve as a good model for BNNs. The artificial neural networks were originally inspired by neural computation and the structure of the brain (Hassabis et al., 2017). Although much of the subsequent development has been made in terms of mathematics and engineering based on efficient optimization rather than neuroscientific findings, there are still opportunities for synergy with neuroscience (Marblestone et al., 2016). Since they share the question of how to analyze the representations of neural networks, data analysis tools and concepts established in each field can facilitate the re-examination of the preconceptions as well as the development of fresh methodologies and theories (Barrett et al., 2019). Among the “AI to neuroscience” approaches, there have been successful attempts to adopt DNNs as an *in silico* model system for BNNs, suggesting testable hypotheses for neural computing (Cohen et al., 2019; Lillicrap and Kording, 2019; Richards et al., 2019; Bellec et al., 2020). This has provided a significant amount of insight into the elucidation of information processing in the brain. Here, we demonstrated that *in silico* simulation using DNNs may also be particularly effective in articulating the influences that the assumptions of a researcher can bring. Compared to the BNNs, the DNNs can be said to be a more explainable model, at least in that they can explicitly demonstrate the results of a strategic manipulation of the assumption or specific factor in a controlled setting, thus enabling us to detect and remove biases more readily (Koh and Liang, 2017; Samek et al., 2017). In the study, we used the fully connected network and the convolutional neural network model to mimic and display the expected error situations in BNN research. However, depending on the research subject and target, researchers may be able to use DNNs with other inductive biases and customize the analysis, structure, and learning of the model.

Lastly, it is worth noting that the simulated scenarios were not mutually exclusive and were only described with emphasis on certain aspects of potential bias. Additionally, the issues presented herein are only illustrative examples that aim to highlight topics of concern regarding the currently adopted research strategy. Although our study did not go so far as to suggest an alternative paradigm, we tried to demonstrate possible biases and constraints in the research to understand neural representation, and we believe that this approach can contribute to encouraging the discussion and efforts to revisit and complement them. In conclusion, by incorporating lessons from the point of contact with the AI field into research experiences in neuroscience, we will be able to gain insights and devise creative approaches to investigating the operating principles of the brain.

## Materials and Methods

### The DNN Trained on CIFAR-10 as a Model of BNN

We constructed the DNN classifier using the open-source neural network library Keras (Chollet, 2017) while operating TensorFlow (Abadi et al., 2016) as the backend. The model comprised six layers with dense connections (rectified linear activation for five hidden layers with 400, 200, 100, 50, and 20 nodes and softmax activation for the output layer with 10 nodes). A dropout layer with a keep probability of 0.8 and an l2-regularizer was added on each layer to apply penalties on the layer activity during optimization. The model was trained with the CIFAR-10 dataset comprising 60,000 32 × 32 color images in 10 classes, with 6,000 images per class, and the dataset was split 75%/8%/17% for the training/validation/test sets. After 500 epochs of training using stochastic gradient descent, the model demonstrated a saturated test set accuracy of ~53% (chance-level performance = 10%).

### Scenario 1. Suboptimal Feature Selectivity

Each class unit in the output layer of the DNN model is treated as a neural unit tuned to the corresponding class, and they calculate the probability of the class for the input image. After feeding each of the 1,000 test set images per class to the trained model, we obtained the output values of the truck unit and regarded them as neural activities of the concept neuron tuned to the truck class. The average outputs per input class were presented as the selectivity profile of the truck unit. The multiple linear regression was implemented to model the relationship between the class features and the responses of the truck unit. We fitted the model with the activation of the truck unit as response variables, and the 10 class features (one-hot vectors) as predictors and presented the coefficients and their *p-*values for the automobile and the truck feature. In addition, we calculated the explained variance statistic of each feature, which can be used as the indicator of statistical effect size.

### Scenario 2. Irrelevant Feature Selectivity

To mimic a situation in which the dataset prepared by the researcher contains an unrecognized confounding variable, two veterinary hospital logo images were synthesized at consistent positions of the cat and dog images (lower right and lower left). The logos were synthesized with 80% transparency after a down-resolution to 5 × 5 × 3, considering the dataset resolution. Subsequently, the accuracy of the trained model in distinguishing the class between the cat and dog was evaluated for both the original and modified (logo-synthesized) datasets.

To expose the implicit attention of the trained model on an image, we tried to visualize the receptive field for each unit in the output layer. Although an analytical solution for the inverse of the feedforward connection cannot be obtained, the degree of contribution to the output unit activation for each input pixel can be estimated using the learned weight parameters. For every route linking a single input pixel and a class unit, from back to front, a one-hot class vector was sequentially multiplied by the weights of the connections and passed through the inverse of the rectified linear activation. In this way, the values obtained from all connecting routes were summed and assigned as the intensity value of the corresponding input pixel, meaning that the pixels with high intensity are discriminative image regions. Then, 3,072-dimensional receptive field vectors were converted into RGB images (32 × 32 × 3) for visualization. To confirm the reliability of the method for reconstructing the receptive field vectors, we asked the trained model to predict the class. The model had correct predictions for 8 out of 10 classes. Considering the possibility that the error was attributed to the incomplete learning (test set accuracy = 53%) of the model, we evaluated the method for the same model trained on the MNIST data. MNIST is a database of handwritten digits comprising a training set of 60,000 images for 10 classes and 10,000 test set images. After training the model (test set accuracy = 92%) and reconstructing the receptive field vectors of the class units in the same manner, the model had correct predictions for all 10 classes.

### Scenario 3. Overestimation of the Network Feature Representation

In BNN research, linear SVM is commonly regarded as a surrogate for a linear read-out neuron, and its decoding performance is used to estimate the amount of information represented in the region of interest. We mimicked the situation in which the researcher evaluates whether the cat feature is represented in a brain region based on the performance of a linear decoder trained to discriminate the feature labels for the neural population activities. The last hidden layer of the DNN model was regarded as the region of interest, and we obtained the output vectors of that layer (treated as recorded population activities) after feeding 1,800 test set images labeled as cat or non-cat. The non-cat images comprised every 100 images of the other 9 classes. The linear SVM was trained to classify the class (cat or non-cat) for those output vectors. Subsequently, the classification accuracy was evaluated on the remaining 100 test set images of cat for both the DNN model and linear SVM.

### Scenario 4. Overestimation of the Noise Correlation

#### Feature Complexity

This scenario mimics the situation in which a researcher repeats multiple trials, recording neural responses to each stimulus presentation. Specifically, it is assumed that in each of *M* trials, a researcher presents a single image from the image set with different subclasses (fine-grained feature) within the same class (coarse-grained feature) and records *k* neurons per each of *N* units with each tuned to the respective class. [The number of trials (*M*), the number of units (*N*), and the number of recorded neurons per unit (*k*) were set to 1,000, 10, and 9 in the simulation, respectively].

We model the *k*-dimensional vector of neurons for each unit as follows:


(1) $$\begin{array}{l} x_{n,m}\;\sim \;N_{k}\left(\mu_{n,m},\;{\sigma_{n,m}}^{2}I\right) \end{array}$$

where *n* = 1, …, N, *m* = 1, …, M, ***x***_*n,m*_ = ${\left(x_{1,n,m}{,\;x_{2,n,m},...,\;x}_{k,n,m}\right)}^{T}\in R^{k}$ denotes the responses of “*k* individual neurons” within the *n*th unit to the *m*th image, with mean vector $\mu_{n,m}\in R^{k}$ and covariance matrix ${\sigma_{n,m}}^{2}I\in R^{k\;x\;k}$.

Here, we set ***μ***_*n,m*_ to μ_*n,m*_ **1**, indicating that all *k* neurons have the same mean.

Subsequently, the distribution of “unit activity” will also be a normal distribution with the same mean but variance divided by sample size (*k*):


(2) $$\begin{array}{l} X_{n,m}\;\sim \;N\left(\mu_{n,m},\;\frac{{\sigma_{n,m}}^{2}}{k}\right) \end{array}$$

where *X*_*n,m*_ denotes the *n*th unit activity to the *m*th image, which represents the within-trial sample mean of the individual neural activities.

However, the values we can observe from the DNN model are the activations of the units, not the individual neurons (it can be slightly confusing because it is different from the general experimental situation). When we feed *M* images with different subclasses of the tuned class, the *n*th unit in the last layer outputs *M* values *X*_*n*,1_, ..., *X*_*n,M*_, which each corresponds to the within-trial sample mean of the *k* recorded neurons. While the noise correlation is defined as the correlation between the response variability of neurons during repeated presentations of identical stimuli, in a trained model, units output a fixed value for the same input. In other words, they do not exhibit any response fluctuation when the same stimulus is repeated (i.e., deterministic). Hence, the parameters of a probability model of individual neurons had to be estimated from the unit activities, enabling stochastic sampling.

In this scenario, we assume two different models with coarse- and fine-grained representations, each of which encodes the class and subclass of an image stimulus, respectively. First, for the coarse-grained representation model, every *M* image with different subclasses is treated as the same stimulus. To generate ***x***_*n,m*_, we estimated the mean vector and the covariance matrix of ***x***_*n,m*_ as follows:


(3) $$\begin{array}{l} \hat{\mu_{n,m}}=X_{n,m}1,\hat{{\sigma_{n,m}}^{2}}=k\cdot {SD\left(X_{n,m}\right)}^{2} \end{array}$$

where **1** ∈ ***R***^*k*^ is the all-ones vector, $\hat{\sigma_{n,m}}$ denotes the within-unit variability of the *n*th unit in the *m*th trial, and *SD* denotes the sample standard deviation.

This estimation makes the standard deviation of the observed *X*_*n,m*_s the standard error of the mean for *k* individual neurons.

Second, for the fine-grained representation model, every *M* image is treated as a distinctive stimulus. The mean vector of ***x***_*n,m*_ is derived in the same way as follows:


(4) $$\begin{array}{l} \hat{\mu_{n,m}}=X_{n,m}1 \end{array}$$

where **1** ∈ ***R***^*k*^ is the all-ones vector.

On the other hand, the within-unit variability is estimated by supposing the variance ratio with the coarse-grained representation model. The variance ratio is set as the theoretical percentile value of the F-distribution to ensure that their ratio is significantly incompatible with the null hypothesis that two independent normal variances are equal:


(5) $$\begin{array}{l} \hat{{\sigma_{n,m}}^{2}}=\frac{1}{F_{0.05,\;k-1,k-1}}\cdot k\cdot {SD\left(X_{n,m}\right)}^{2} \end{array}$$

where *F*_0.05, *k*−1, *k*−1_ is the critical value from the F-distribution with *k-1* and *k-1* degrees of freedom such that the probability of erroneously rejecting the null hypothesis is 5%.

This ensures that the within-unit variability of the fine-grained model is much smaller than that of the coarse-grained model such that the difference in the responses to the subclasses is significant for the former and non-significant for the latter.

The actual simulation using the model was conducted in two stages. First, we conducted 1,000 trials for each unit, presenting the respective subclass image of the class in which the unit is tuned. In each trial, the within-unit variability ($\hat{{\sigma_{n,m}}^{2}})$ for both the coarse- and fine-grained models are obtained for each unit. Afterward, while presenting 1,000 “dog” images with different breeds, the activation values were extracted from all 10 class units. From this, the sample mean of the individual neural responses to the respective dog image is estimated for each unit ($\hat{\mu_{n,m}})$. Hereby, the multivariate normal distribution of individual neural activities for the respective dog image can be estimated from the obtained sample mean and the within-unit variability. For each model, the pairwise Pearson correlations were calculated between the trial-by-trial variability of sampled neurons and represented in a correlation matrix.

#### Internal Dynamics

Here, we tried to show that if a periodic internal rhythm is globally represented in the neural population, the resulting co-fluctuation of neural excitability can inflate the estimates of the noise correlation. Simplifying the situation, we assumed that the phases in which the neural excitability increases or decreases (referred to as upcycle or downcycle) alternate in each trial.

As a control, we employed the multivariate normal distribution of the coarse-grained model in scenario 4.1, and the activities of *k* neurons per each of *N* unit were sampled therefrom. For the model assumed to encode a periodic internal rhythm, we randomly generated values from the uniform distribution over (0, 1) and added them to the unit outputs for odd-numbered trials and subtracted them from the outputs for even-numbered trials. The within-unit variabilities were set to be identical to those of the control distribution. After estimating the parameters of the distribution of *k* neurons per unit in the same way as before, the individual neural activities in 1,000 trials were sampled. For each model, the pairwise Pearson correlations between the trial-by-trial variability of the sampled neurons were calculated and represented in a correlation matrix.

### Reproducing the Scenarios Using a CNN

We used the pretrained ResNet18 model provided by the PyTorch library (Paszke et al., 2019) and fine-tuned the model for the cifar-10 dataset for 3 epochs with a batch size of 40. All analyses were performed in the same manner as in the fully connected network model except for the feature map visualization for scenario 2, where we exploited the class activation map method (Zhou et al., 2016).

## Data Availability Statement

The original contributions presented in the study are included in the article/Supplementary Materials, further inquiries can be directed to the corresponding author/s.

## Author Contributions

HB, C-EK, and SK: conceptualization. HB: investigation and writing—original draft. C-EK and SK: writing—review & editing and funding acquisition. All authors contributed to the article and approved the submitted version.

## Conflict of Interest

The authors declare that the research was conducted in the absence of any commercial or financial relationships that could be construed as a potential conflict of interest.
