# Erratum: Lessons From Deep Neural Networks for Studying the Coding Principles of Biological Neural Networks

**DOI:** 10.3389/fnsys.2022.977573

**Published:** 2022-08-17

**Authors:** Frontiers Production Office

**Affiliations:** Frontiers Media SA, Lausanne, Switzerland

**Keywords:** deep neural networks, biological neural networks, systems neuroscience, shortcut learning, neural coding, neural feature

Due to a production error, there was a mistake in Figure 5B as published. Figure 5D was superimposed over Figure 5B. The corrected Figure 5B appears below.

**Figure 5:**
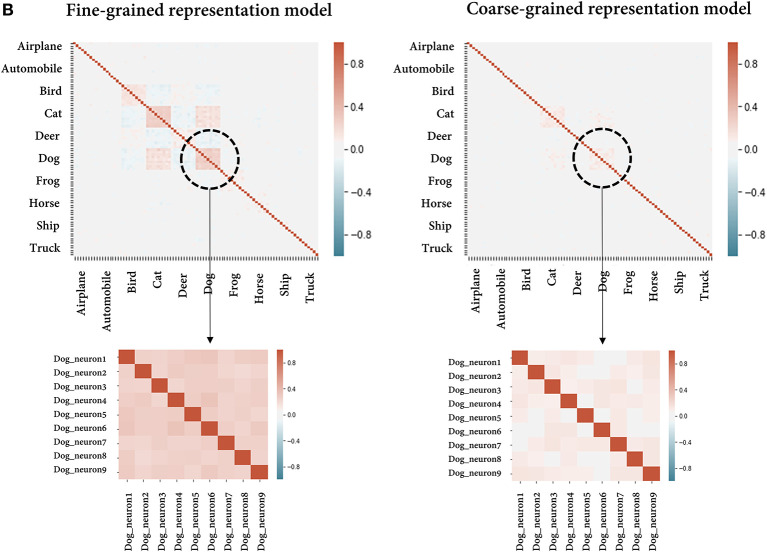


The publisher apologizes for this mistake. The original version of this article has been updated.

