# Supplementary material for: Lessons From Deep Neural Networks for Studying the Coding Principles of Biological Neural Networks
Source: Front Syst Neurosci. 2021 Jan 15;14:615129. doi: 10.3389/fnsys.2020.615129 (PMC7843526; doi:10.3389/fnsys.2020.615129)
Supplement: Supplementary file 1 [file Table_1.DOCX]

Supplementary Material

## Supplementary Figures


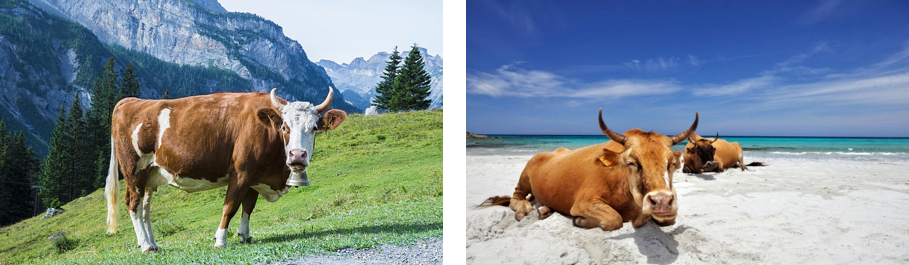


**Supplementary figure 1. Examples of shortcut learning in deep learning**. While the deep neural network can accurately classify the image when the primary object is in a ‘common’ context (left), it unexpectedly fails when the primary object is in an ‘uncommon’ context (right) (each image is from <https://www.pickpik.com> and [www.freeimages.co.uk](http://www.freeimages.co.uk), respectively).


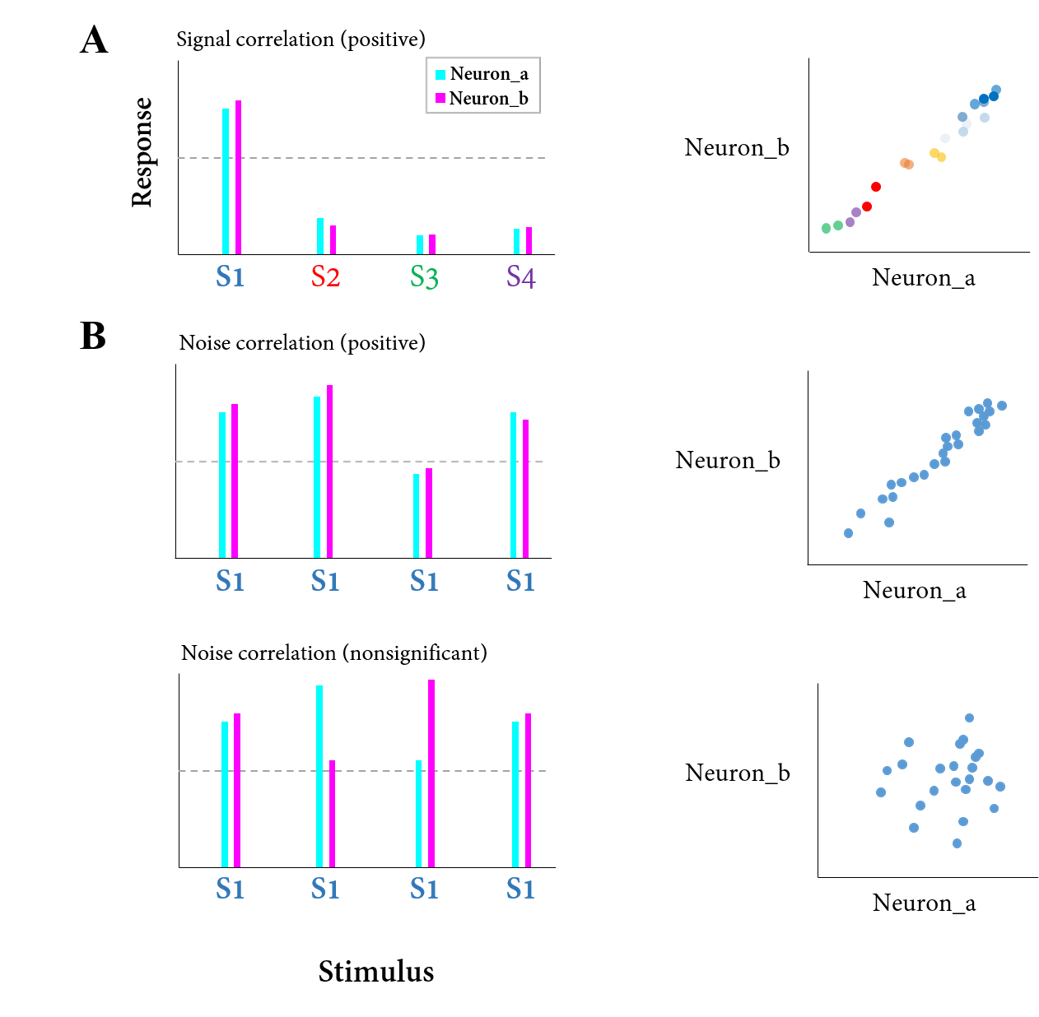


**Supplementary figure 2. A Schematic figure illustrating the signal and noise correlation**. (A) Signal correlation. The pairwise correlation between neural responses over an ensemble of stimuli has commonly been used to refer to a similar tuning property. (B) Noise correlation. The pairwise correlation between neural responses upon repeated presentation of the identical stimulus measures the extent to which the trial-by-trial variability in response strength is shared. S1 to S4 in the bar graph denote each given stimulus, and each point in the scatter plot represents the mean response of the pair of neurons to a given stimulus (represented in color). Although not shown in the figure, both the signal and noise correlations can be negative.
